# An analysis of the ArcCHECK‐MR diode array's performance for ViewRay quality assurance

**DOI:** 10.1002/acm2.12107

**Published:** 2017-06-06

**Authors:** Steven T. Ellefson, Wesley S. Culberson, Bryan P. Bednarz, Larry A. DeWerd, John E. Bayouth

**Affiliations:** ^1^ Department of Radiation Oncology Mayo Clinic Arizona Phoenix AZ USA; ^2^ School of Medicine and Public Health Department of Medical Physics University of Wisconsin‐Madison Madison WI USA; ^3^ School of Medicine and Public Health Department of Human Oncology University of Wisconsin‐Madison Madison WI USA

**Keywords:** ArcCHECK, diode array, IMRT quality assurance, MR‐IGRT, ViewRay

## Abstract

The ArcCHECK‐MR diode array utilizes a correction system with a virtual inclinometer to correct the angular response dependencies of the diodes. However, this correction system cannot be applied to measurements on the ViewRay MR‐IGRT system due to the virtual inclinometer's incompatibility with the ViewRay's multiple simultaneous beams. Additionally, the ArcCHECK's current correction factors were determined without magnetic field effects taken into account. In the course of performing ViewRay IMRT quality assurance with the ArcCHECK, measurements were observed to be consistently higher than the ViewRay TPS predictions. The goals of this study were to quantify the observed discrepancies and test whether applying the current factors improves the ArcCHECK's accuracy for measurements on the ViewRay. Gamma and frequency analysis were performed on 19 ViewRay patient plans. Ion chamber measurements were performed at a subset of diode locations using a PMMA phantom with the same dimensions as the ArcCHECK. A new method for applying directionally dependent factors utilizing beam information from the ViewRay TPS was developed in order to analyze the current ArcCHECK correction factors. To test the current factors, nine ViewRay plans were altered to be delivered with only a single simultaneous beam and were measured with the ArcCHECK. The current correction factors were applied using both the new and current methods. The new method was also used to apply corrections to the original 19 ViewRay plans. It was found the ArcCHECK systematically reports doses higher than those actually delivered by the ViewRay. Application of the current correction factors by either method did not consistently improve measurement accuracy. As dose deposition and diode response have both been shown to change under the influence of a magnetic field, it can be concluded the current ArcCHECK correction factors are invalid and/or inadequate to correct measurements on the ViewRay system.

## 1 INTRODUCTION

Magnetic resonance imaging‐guided radiation therapy has tremendous potential for real‐time image guidance during treatment delivery. The ViewRay system (ViewRay, Inc., Oakwood Village, OH, USA) is the world's first MRI‐guided delivery system, and uses a ring gantry with three cobalt‐60 (^60^Co) treatment heads mounted 120° apart, each with an independent double‐focused multileaf collimator (MLC).^1^ The system is capable of delivering a variety of treatment options, including adaptive and intensity‐modulated radiation therapy, all with simultaneous image guidance using its integrated 0.35 T MRI system. Given the novelty and complexity of this new system, robust delivery verification procedures must be developed and tested to ensure accurate treatment delivery. The ViewRay system presents a unique challenge to IMRT QA devices due to its ability to deliver up to three simultaneous beams in the presence of a significant magnetic field.

The ArcCHECK device (Sun Nuclear Corporation, Melbourne, FL, USA) is a helical diode array consisting of 1386 diodes encased within a cylindrical polymethyl methacrylate (PMMA) shell at a water‐equivalent depth of 3.3 g cm^−2^ (physical depth of 2.9 cm). The ArcCHECK‐MR model is one of the few IMRT QA devices with claimed MRI‐compatibility up to magnetic field strengths of 0.35 T. This is accomplished by placing the device's power source outside the 5 gauss line. To correct the known angular response dependencies of the diodes, the ArcCHECK software utilizes a correction system coupled with a virtual inclinometer algorithm.^2^ The algorithm analyzes the distribution of measured signals within the ArcCHECK, determines the angle of the incident beam for every 50 ms measurement interval, and applies predetermined correction factors. The virtual inclinometer algorithm has previously been shown to be accurate within 1°. Previous studies of the ArcCHECK system by Kozelka et al. and Li et al. found it to have an accuracy and precision acceptable for clinical IMRT and VMAT quality assurance.^2^, ^3^ However, it must be noted that these studies did not use the MRI‐compatible ArcCHECK and all measurements were performed without the presence of a significant magnetic field.

Despite the success of the ArcCHECK's correction system coupled with its virtual inclinometer algorithm, the correction system is unable to be utilized for measurements on the ViewRay system, as the algorithm assumes there is only a single incident beam in any 50 ms time interval. Consequently, correction factors are not currently applied to ArcCHECK measurements performed for patient‐specific quality assurance on the ViewRay at our institution. Additionally, the current correction factors were determined without magnetic field effects taken into account. Dose deposition at interfaces of materials with different stopping powers, such as those found near diodes in the ArcCHECK, and diode response have been shown to change under the influence of magnetic fields.^4^, ^5^, ^6^, ^7^ As the correction factors are determined by the diodes' responses, it is possible the current factors are not valid for measurements within the ViewRay's 0.35 T magnetic field.

All manufacturer‐recommended procedures for maximizing agreement between the ArcCHECK and the ViewRay TPS were followed during commissioning of the ArcCHECK at our institution. This includes adjusting the electron density of the ArcCHECK image dataset in the ViewRay TPS until 3%/3 mm gamma passing rates are near 100% for reference treatment plans. Maximum agreement between the ViewRay TPS and the ArcCHECK at our institution was achieved with an electron density value of 1.125 g cm^−3^.

In the course of performing patient‐specific quality assurance measurements with the ArcCHECK for the first patients treated on the ViewRay at our institution, it was observed the ArcCHECK measurements were consistently higher than the ViewRay TPS predictions. Accordingly, the goals of this study were to quantify the observed discrepancies and test whether applying the current correction factors improves the ArcCHECK's accuracy for measurements on the ViewRay system.

## 2 METHODS

### 2.A Quantify discrepancies

**Figure 1:**
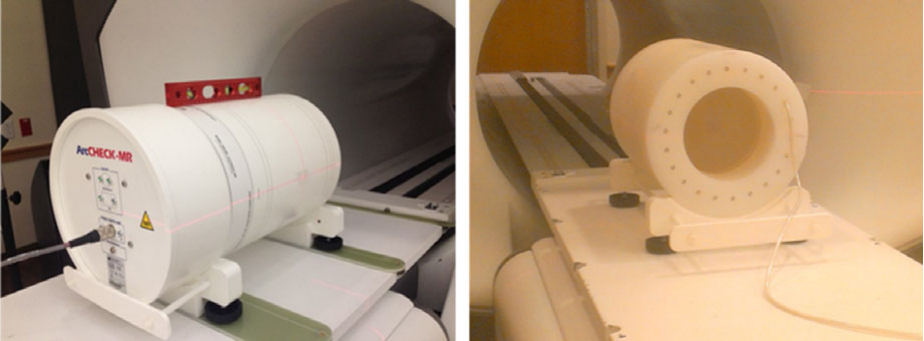
Pictures of the patient‐specific QA setup for the ArcCHECK (left) and the experimental ionization chamber phantom setup (right) in the ViewRay system.

To quantify the discrepancies between the ViewRay TPS and ArcCHECK measurements, 19 ViewRay patient plans were analyzed. Gamma analysis was performed using the SNC Patient software using a 10% global threshold.^8^ Analysis was performed with parameters set at 3%/3 mm, 2%/2 mm, and 1%/1 mm. The “Apply Measurement Uncertainty” option in the SNC Patient software was used for the ArcCHECK measurements. Frequency analysis on the differences between the ViewRay TPS and ArcCHECK measurements was also performed. Ten diode measurements in high dose, low gradient regions within their respective plans were then chosen to compare with Accredited Dosimetry Calibration Laboratory (ADCL)‐calibrated Exradin A1SL ionization chamber (Standard Imaging, Middleton, WI, USA) measurements in the same locations. Each of the chosen diode measurements failed gamma analysis at 3%/3 mm. Ion chamber measurements were performed in a phantom with the same size and shape as the ArcCHECK, provided by the Sun Nuclear Corporation (see Fig. 1). The manufacturer claimed that the phantom is composed of PMMA. In place of diodes, the phantom has holes drilled in it to fit Exradin A1SL ion chambers, with PMMA plugs to fill the holes not being used. The holes are drilled such that ion chamber measurements can be taken at locations corresponding to diodes in the ArcCHECK. Measurements with the ion chamber phantom were performed with the ArcCHECK's central PMMA plug inserted into the phantom's central cavity.

The ViewRay treatment planning system and the ArcCHECK report dose‐to‐water. To acquire the dose‐to‐water for ion chamber measurements in the PMMA phantom, a modified form of the dose formalism presented by Seuntjens et al. was used in this work.^9^ The Seuntjens et al. formalism relates measurements made in solid nonwater phantoms to the absorbed dose‐to‐water that would be obtained using a water phantom and the AAPM's Task Group 51 (TG‐51) protocol. The dose‐to‐water at a defined depth *z*_ref_ from an ion chamber measurement in a PMMA phantom is given by

(1) $$\begin{array}{cc} D_{w}\left(z_{\mathrm{ref}}\right)=\; & M_{\mathrm{raw}}P_{\mathrm{pol}}P_{\mathrm{elec}}P_{\mathrm{TP}}P_{\mathrm{MF}}N_{D,w}^{\mathrm{Co}-60}k_{Q}\\ & \left[\frac{D_{w}\left(z_{\mathrm{ref}}\right)}{D_{\mathrm{PMMA}}\left(z_{\mathrm{eq}}\right)}\frac{{\left(\frac{\overset{¯}{L}}{\rho }\left(z_{\mathrm{eq}}\right)\right)}_{\mathrm{air}}^{\mathrm{PMMA}}}{{\left(\frac{\overset{¯}{L}}{\rho }\left(z_{\mathrm{ref}}\right)\right)}_{\mathrm{air}}^{w}}{\left(P_{\mathrm{ion}}P_{\mathrm{wall}}\right)}_{w}^{\mathrm{PMMA}}\right], \end{array}$$

where *M*_raw_ is the raw charge measurement, *P*_ion_, *P*_pol_, *P*_elec_, and *P*_TP_ are the standard corrections used for reference dosimetry of high‐energy photon beams, *k*_Q_ is the “beam quality factor” which converts the calibration coefficient to the beam of interest, and $N_{D,w}^{\mathrm{Co}-60}$ is the ADCL calibration coefficient for dose to water from a ^60^Co beam.^11^ For this work, ion recombination and polarity corrections were not applied but expected ranges for the factors from published data were incorporated into the uncertainty budget for the measurements (see Table 1).

**Table 1:** Uncertainty budget for ionization chamber measurements

Source	Uncertainty (%, k = 1)	Comments
Measurement	0.5	Based on variation in repeated isocenter measurements
*P*_pol_	0.3	Maximum allowed deviation for reference class ionization chamber 11
*P*_ion_	0.3	Rounded up estimate based on recombination for ^60^Co ADCL calibration and data from ViewRay commissioning with same model of chamber; assumed rectangular distribution with M = 0.5%
*P*_TP_	0.1	Rounded up from uncertainty due to measured variations in pressure and temperature throughout measurement period
*P*_elec_	0.1	0.2% at k = 2 reported by ADCL Report
*P*_MF_	1.0	Estimate based on literature 12, 13, 14
$N_{D,w}^{\mathrm{Co}-60}$	0.7	1.4% at k = 2 reported by ADCL Report
*k*_Q_	0.5	Based on variations in *k*_Q_ listed in TG‐51^11^
Small‐field effects	2.0	Estimate based on ion chamber under‐responses to small fields reported in literature^21^, ^22^
Chamber position	0.5	Estimate based on uncertainty in placement and dose gradient in treatment plan
Chamber volume averaging	0.8	Estimate based on total gradient in chamber volume; used rectangular distribution with M = 2%
${\left(\frac{\overset{¯}{L}}{\rho }\right)}_{w}^{\mathrm{PMMA}}$	1.4	Estimate based on uncertainties reported in literature^10^, ^23^
$\frac{D_{w}\left(z_{\mathrm{ref}}\right)}{D_{\mathrm{PMMA}}\left(z_{\mathrm{eq}}\right)}$	0.2	Based on agreement between measurements and calculations reported by Seuntjens et al.^9^
Total	3.0%	

For measurements within the ViewRay's 0.35 T magnetic field, it is necessary to include an additional factor which accounts for magnetic field effects to the chamber's response, notated *P*_MF_ in eq. (1). It has been shown that while the dose distribution from a high‐energy photon beam in a homogeneous medium is only slightly affected by the presence of a magnetic field, there is a large impact on the dose distribution at tissue‐air interfaces due to the altered point spread kernel of secondary electrons.^6^, ^7^, ^12^ As an ion chamber is a gas‐filled cavity, its response in an otherwise homogeneous medium is dependent on the strength and orientation of the magnetic field in a way that the medium's dose distribution is not. The change in a chamber's response is dependent on many factors, including the magnetic field strength, presence and size of air gaps around the chamber, chamber design, and orientation of the chamber with respect to the magnetic field.^5^, ^12^, ^13^, ^14^ A characterization of the A1SL's response within the ViewRay environment has not yet been published. However, literature suggests the potential response dependency of chambers similar to the A1SL is 1% or less.^13^, ^14^, ^15^ For this work, a correction factor was not applied, but the potential response dependency was incorporated into the uncertainty budget for the measurements.

It is important to note *k*_Q_ and $N_{D,w}^{\mathrm{Co}-60}$ are defined for the specific TG‐51 measurement setup of a 10 cm × 10 cm field and depth of 10 cm in a rectangular water phantom.^11^ As the experimental setup differs significantly from these conditions, there is additional uncertainty in the use of each of the factors due to possible changes in the scatter conditions around the chamber. *k*_Q_ was assumed to be 1.000 for this work, as defined by TG‐51 for ^60^Co beams, and estimates of the uncertainty for each factor were derived from literature and incorporated into the experiment's uncertainty budget in Table 1.

As the ion chamber phantom material is PMMA and not water, it is necessary to apply additional corrections to the ion chamber measurements in order to obtain the absorbed dose‐to‐water.^9^ These corrections are contained in the bracketed expression in eq. (1). $D_{w}\left(z_{\mathrm{ref}}\right)/D_{\mathrm{PMMA}}\left(z_{\mathrm{eq}}\right)$ is the ratio of dose‐to‐water at the defined depth to the dose‐to‐PMMA at the corresponding water‐equivalent depth. ${\left(\frac{\overset{¯}{L}}{\rho }\left(z_{\mathrm{ref}}\right)\right)}_{\mathrm{air}}^{w}$ is the ratio of restricted stopping powers between water and air at the specified depth, and ${\left(\frac{\overset{¯}{L}}{\rho }\left(z_{\mathrm{eq}}\right)\right)}_{\mathrm{air}}^{\mathrm{PMMA}}$ is the ratio between PMMA and air at the water‐equivalent depth. *P*_wall_ is the factor used to correct for changes in the electron spectrum within the chamber volume caused by the chamber wall.^20^

$D_{w}\left(z_{\mathrm{ref}}\right)/D_{\mathrm{PMMA}}\left(z_{\mathrm{eq}}\right)$ may be determined via Monte Carlo simulations or use of the scaling theorem.^9^ For this work, we used the Monte Carlo‐derived value of 0.986 for ^60^Co in PMMA reported by Seuntjens et al.^9^ For measurements taken in locations at or deeper than *d*_max_, the restricted stopping power ratio for a ^60^Co beam does not depend on the depth of measurement.^10^ For this work, we used the ratios reported by Seuntjens et al., which give a value for ${\left(\frac{\overset{¯}{L}}{\rho }\right)}_{w}^{\mathrm{PMMA}}$ of approximately 0.973. As the ratio of *P*_wall_ factors for the A12 chamber between PMMA and water reported by Seuntjens et al. is 1.000 and the A1SL chamber has the same wall material as the A12, this factor was assumed to be unity for this work. The ratio of *P*_ion_ values between PMMA and water reported by Seuntjens et al. is close to unity. As we did not apply a *P*_ion_ correction to our measurements, but incorporated the factor's expected range into our uncertainty budget, the difference in *P*_ion_ between measurements in water and PMMA is within our stated uncertainty. With all of these considerations taken into account, eq. (1) simplifies to

(2) $$D_{w}\left(z_{\mathrm{ref}}\right)=M_{\mathrm{raw}}P_{\mathrm{elec}}P_{\mathrm{TP}}N_{D,w}^{\mathrm{Co}-60}\left(\frac{D_{w}\left(z_{\mathrm{ref}}\right)}{D_{\mathrm{PMMA}}\left(z_{\mathrm{eq}}\right)}\right){\left(\frac{\overset{¯}{L}}{\rho }\right)}_{w}^{\mathrm{PMMA}},$$

with all of the known dependencies taken into account in the uncertainty budget for the measurements.

To validate the experimental method, additional patient plan measurements were taken, consisting of isocenter ion chamber measurements at the center of both the ArcCHECK and the ion chamber phantom and at a point where the ArcCHECK and ViewRay TPS agree within 1%. These measurements were performed to verify the dose calibration of the ArcCHECK.

### 2.B Test current correction factors

In order to analyze the current ArcCHECK correction factors, it was necessary to develop a method of applying the factors to ArcCHECK measurements of ViewRay plans. As the ArcCHECK's virtual inclinometer is not compatible with multiple simultaneous beams, the new method was required to derive beam information an alternate way. As the beam delivery angles for each plan are known by the ViewRay system, this was accomplished by exporting beam information from the ViewRay TPS to the correction algorithm. As the ViewRay system at our institution is kept within the tolerances recommended by the AAPM Task Group 142 report, the delivery angles reported by the ViewRay system should have comparable accuracy to the virtual inclinometer algorithm.^2^, ^16^

As correction factors for an arbitrary beam angle must be applied to the measured diode signal specifically from that beam, before applying correction factors to ArcCHECK diode measurements it was necessary to weight the applied factors by the proportion of total dose to each diode from each beam. Using the ArcCHECK measurement and correction paradigm detailed by Kozelka et al. and the Sun Nuclear Corporation's ArcCHECK reference guide and accounting for dose proportion from each beam, the total corrected dose to diode *i* is

(3) $$D_{\mathrm{TOT},\mathrm{corr},i}=\frac{D_{\mathrm{TOT},\mathrm{uncorr},i}}{\sum_{k=1}^{K}\frac{F_{i,k}}{C_{\mathrm{AD},i,k}C_{\mathrm{ID},i,k}C_{\mathrm{FS},i,k}C_{\mathrm{HF},i,k}}},$$

where *F*_i,k_ is the fraction of dose to diode *i* from beam *k* and the *C* factors are the four correction factors currently used for ArcCHECK measurements, correcting for dependencies on angular incidence, variation between individual diodes, field size, and phantom heterogeneity. The derivation of eq. (3) is given in Appendix A. eq. (3) was implemented in MATLAB 2015a (The MathWorks, Inc., Natick, MA, USA). Expected dose fraction values for each beam were calculated using DICOM RT Dose files for each beam in a plan. The correction factors utilized were provided by the Sun Nuclear Corporation and were reported to have been derived for ^60^Co beams. To ensure the new method references and applies the correction factors properly, portions of MATLAB code from a research version of the Sun Nuclear Corporation's correction algorithm were used or altered to work with the new method. The research version of the Sun Nuclear Corporation's correction algorithm was provided by the Sun Nuclear Corporation, and will herein be referred to as the “SNC algorithm”.

It must be noted the purpose of the new method is to apply directionally dependent correction factors and thus serve as an alternative to the ArcCHECK's virtual inclinometer algorithm. The field size correction factor is distinct from the other three factors in that it is dependent on the size and shape of the incident beam.^2^, ^17^ The field size correction requires another algorithm, distinct from the virtual inclinometer, to calculate the field size. The issue is even more complicated for deliveries on the ViewRay system, as the ViewRay has three beams, each with its own double‐focused MLC, and may operate in two‐ or three‐head mode. As the field size correction is dependent on the scatter conditions within the phantom, which change depending on the number and shape of beams incident on the ArcCHECK, the applied factors depend on the mode the ViewRay is operating in. Accordingly, the field size correction was not implemented in this work. A comparison of an ArcCHECK measurement without applied corrections and with all corrections applied, including the field size correction, was performed to see the effect of not including the field size correction.

To test the current ArcCHECK correction factors for measurements in the ViewRay environment, the following “Single Beam Experiment” was performed. ArcCHECK measurements were acquired on nine ViewRay patient plans. The measured plans were a subset of the 19 plans analyzed previously. The delivered doses and beam characteristics of the ViewRay plans were unaltered. However, each individual patient plan was separated into three different plans, with each new plan consisting of the original plan's delivery from a single treatment head. ArcCHECK measurements were acquired with each single‐head plan and combined using the SNC Patient software. This enabled use of the ArcCHECK's virtual inclinometer algorithm. The research version of the SNC algorithm was then used to apply corrections after measurement. The field size correction was not applied, to allow for direct comparison with results from the new algorithm.

Corrections were also applied to the Single Beam measurements using the new algorithm. As in Section 2.A, gamma and frequency analysis were performed on the three data sets (uncorrected, SNC corrected, and new algorithm corrected) in comparison with the ViewRay TPS predictions. The gamma analysis parameters were identical to those used in Section 2.A.

In addition to the Single Beam Experiment, the new algorithm was used to apply corrections to the ArcCHECK measurements of all 19 ViewRay patient plans used in the experiment in Section 2.A. The results were compared to the ion chamber measurements, uncorrected ArcCHECK measurements, and ViewRay TPS predictions from Section 2.A. Frequency and gamma analysis were also performed, comparing the new algorithm's results to the ViewRay TPS predictions.

The potential contribution of leakage to the observed discrepancies was analyzed by comparing the results of the Single Beam deliveries to the normal ViewRay deliveries. If leakage is a significant contributor to the observed discrepancies, the magnitude of the discrepancies should be significantly different between the Single Beam and normal ViewRay deliveries, because the Single Beam measurements took approximately three times as long.

To verify both algorithms were properly implemented in MATLAB, a ViewRay plan was also measured in Single Beam mode with the ArcCHECK's corrections mode activated. This is the standard mode used to apply corrections to clinical measurements. The MATLAB implementation of the SNC algorithm was also applied on the same raw measurement, and the two distributions were compared. The beam angles determined by the ArcCHECK's virtual inclinometer were also verified to agree with those stated by the ViewRay system within 1°.

## 3 RESULTS AND DISCUSSION

**Figure 2:**
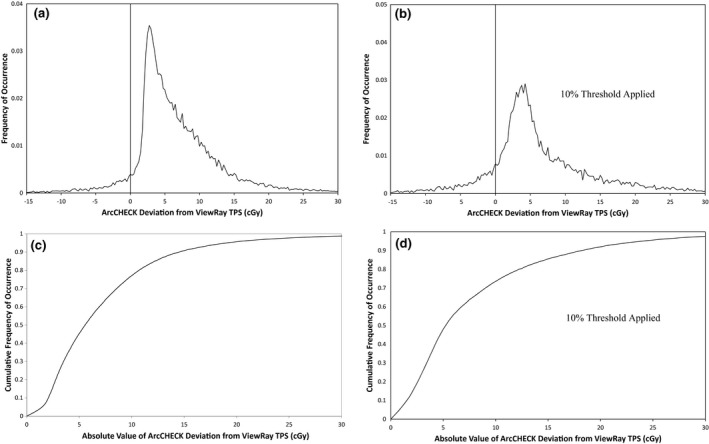
Frequency of occurrence vs. absolute difference in cGy (a and b) and cumulative frequency of occurrence vs. absolute difference in cGy (c and d) between ArcCHECK and ViewRay TPS for 26,334 ArcCHECK diode measurements across 19 ViewRay patient plans. Only measurements meeting a global 10% threshold within their respective plans are analyzed in (b) and (d).

**Figure 3:**
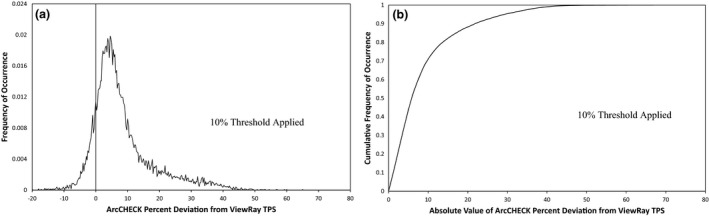
Frequency of occurrence (a) and cumulative frequency of occurrence (b) vs. percent difference between ArcCHECK and ViewRay TPS for ArcCHECK diode measurements across 19 ViewRay patient plans meeting a 10% global threshold.

The gamma analysis results for the ArcCHECK measurements of 19 ViewRay patient plans are displayed in Table 2. The results show that for percent difference and DTA parameters set to 3%/3 mm or higher, good agreement is seen between the treatment plans and ArcCHECK measurements, even without the ArcCHECK corrections applied. However, the average pass rate is much lower and much more variable at 2%/2 mm. This indicates that under the current measurement setup, the ArcCHECK is unable to consistently achieve the same level of agreement with the ViewRay TPS that it has achieved on other systems.^2^, ^3^ Figure 2 shows frequency and cumulative frequency plots of the differences in cGy between the ViewRay TPS and ArcCHECK measurements for the same 19 patient plans, both with and without a 10% global threshold applied. Figure 3 shows the frequency and cumulative frequency plots of the percent difference between the ViewRay TPS and ArcCHECK for measurements meeting a 10% global threshold. For a random measurement uncertainty or error process, Figs. 2(a) and/or 3(a) would be expected to show Gaussian distributions centered about zero.^18^, ^19^ However, it can be seen there is a clear positive offset, meaning the ArcCHECK is consistently reporting doses higher than the ViewRay TPS.

**Table 2:** Average gamma passing rates for ArcCHECK measurements of 19 ViewRay patient plans

	3%/3 mm	2%/2 mm	1%/1 mm
Average gamma pass rate ± 1*σ*, %	96.9 ± 6.8%	84.0 ± 17.5%	49.1 ± 20.2%

In Figs. 2(c) and 2(d), it can be seen the 50% cumulative frequency point is located at differences of approximately 6 cGy for all of the measurements, and at 5 cGy for the measurements meeting a 10% global threshold. This means that half of all of the ArcCHECK measurements on the ViewRay system disagree with the TPS predictions by at least 6 cGy and applying the 10% threshold only slightly reduces this value. In Fig. 3, it can be seen the 50% cumulative frequency point is at a difference of approximately 5%. Using a global definition for the 2% gamma analysis parameter, a 5% local difference will pass gamma analysis only for diodes measuring doses that are at least 40% of the maximum measured dose. Combined with the fact that 5 cGy is 2.5% of a 2 Gy per fraction plan, it is not surprising many of the plans fail gamma analysis at 2%/2 mm.

**Figure 4:**
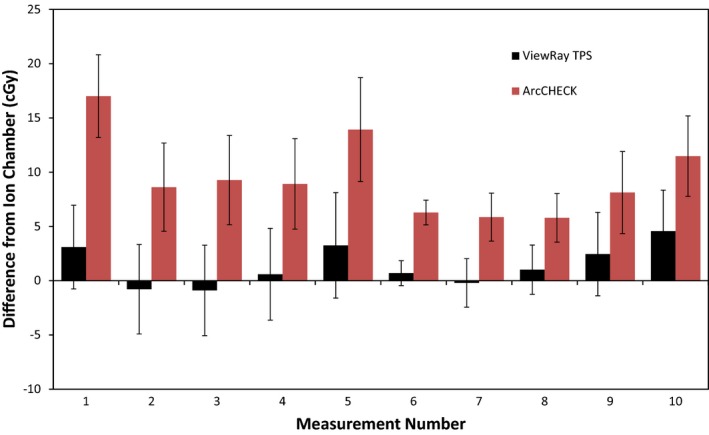
Bar graph depicting differences between ion chamber measurement and ViewRay TPS calculation (black) and ArcCHECK measurement (red). Error bars are representative of total k = 1 measurement uncertainty, derived using the uncertainty budget in Table 1.

Figure 4 and Table 3 display the results of the ion chamber measurements. It can be seen that the ViewRay TPS's average difference from the delivered dose, as measured by the ion chamber, is within the measurement uncertainty. The ViewRay TPS agrees with the ion chamber within uncertainty for nine out of the ten measurement locations. However, for all 10 measurement locations, the ArcCHECK measurements are significantly higher than the ion chamber, and the differences cannot be attributed to measurement uncertainty.

**Table 3:** Average difference from ion chamber for ViewRay TPS and ArcCHECK at ten measurement points

	ViewRay TPS average difference from ion chamber ± 1*σ*	ArcCHECK average difference from ion chamber ± 1*σ*
Difference in cGy	1.4 ± 1.8 cGy	9.5 ± 3.5 cGy
Difference in %	1.3 ± 1.4%	9.3 ± 3.4%

Using a one‐sided t‐test, the calculated *P*‐value for the differences between ArcCHECK comparisons to the ion chamber and ViewRay TPS comparisons to the ion chamber is less than 0.00001. Defining statistical significance at *P *< 0.05, the ArcCHECK's average difference from the ion chamber is therefore significantly different than the ViewRay TPS's average difference.

The isocenter and “agreement point” ion chamber measurements agreed with the ArcCHECK and the ViewRay TPS within approximately 1%, indicating that the experiment's methodology is valid and the observed discrepancies are not due to a miscalibration of the ArcCHECK.

From these results, it can be concluded the ArcCHECK systematically reports doses higher than those predicted by the ViewRay TPS. The ViewRay TPS consistently agrees with ion chamber measurements. Combined with the recent work by Wooten et al., demonstrating the ViewRay system performs within the confidence limits recommended by the AAPM's Task Group 119 Report, it can be concluded that these discrepancies are correlated with the ArcCHECK.^15^

**Figure 5:**
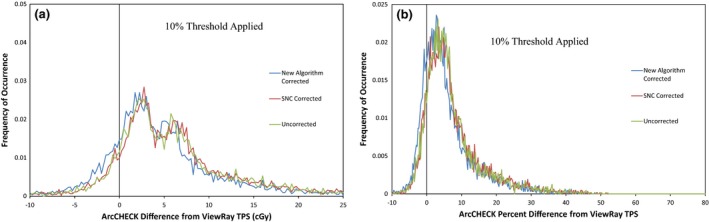
Frequency of occurrence vs. absolute difference in cGy (a) and percent difference (b) between ArcCHECK (uncorrected, SNC corrected, and new algorithm corrected) and ViewRay TPS for ArcCHECK diode measurements across nine ViewRay patient plans delivered in Single Beam mode and meeting a 10% global threshold.

**Figure 6:**
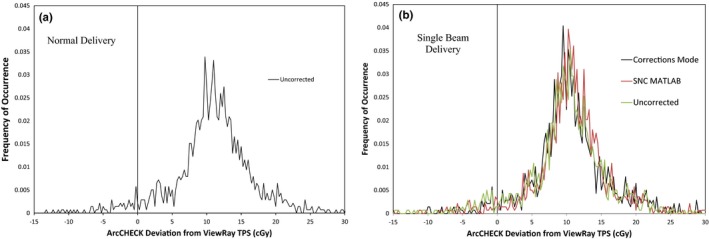
Frequency of occurrence vs. absolute difference in cGy between ArcCHECK (uncorrected, in “corrections mode”, and corrected with the MATLAB implementation of the SNC algorithm) and ViewRay TPS for 1,386 ArcCHECK diode measurements from one ViewRay patient plan delivered in normal mode (a) and Single Beam mode (b).

**Figure 7:**
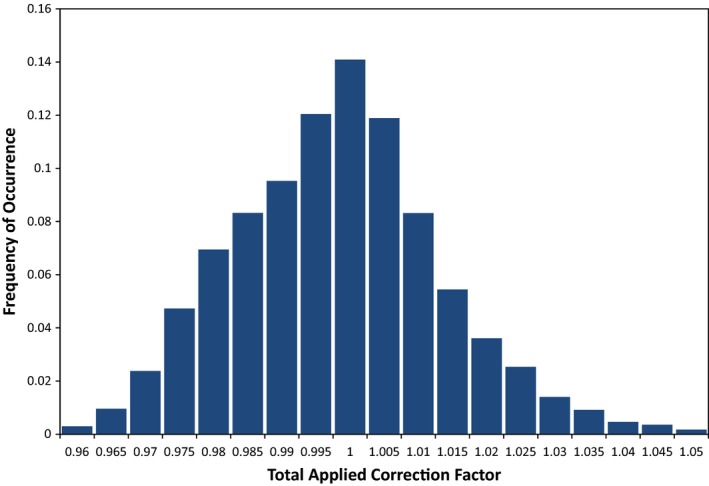
Frequency of occurrence vs. total applied correction factor for 26,334 ArcCHECK diode measurements from 19 ViewRay patient plans. Correction factors applied were the standard corrections used for the ArcCHECK, which were predetermined by the Sun Nuclear Corporation, weighted by the planned dose from each beam to each individual diode.

**Figure 8:**
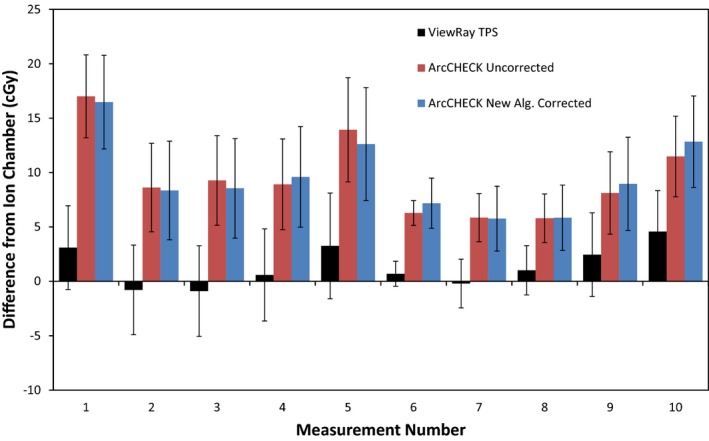
Bar graph depicting differences between ion chamber measurement and ViewRay TPS calculation (black), uncorrected ArcCHECK measurement (red), and ArcCHECK measurement corrected with new algorithm. Error bars are representative of total k = 1 measurement uncertainty, derived using the uncertainty budget in Table 1.

The results of the Single Beam Experiment are given in Table 4 and Figs. 5 and 6, and the results from applying corrections on the original ArcCHECK measurements are given in Tables 5 and 6 and Figs. 7, 8 and 10 Additional factors contributing to the uncertainty of the measurement comparisons are displayed in Table 7. It can be seen in the Single Beam Experiment results that applying the corrections with the new method consistently improved agreement between ArcCHECK measurements and the corresponding ViewRay patient plans. Using a two‐sided t‐test with the gamma analysis results in Table 4, a statistically significant *P*‐value of 0.004 is calculated for the differences between the New Algorithm Corrected and Uncorrected 1%/1 mm datasets. However, at 3%/3 mm, the calculated *P*‐value is 0.28 and is therefore not significant. It is also interesting to note that the spread in passing rates is significantly lower with the new algorithm compared to both the SNC corrected and uncorrected datasets.

**Table 4:** Average gamma passing rates for ArcCHECK measurements from the Single Beam Experiment

	Average gamma pass rate ± 1*σ*, %		
	Uncorrected	SNC corrected	New Alg. corrected
3%/3 mm	98.6 ± 3.8	98.8 ± 3.4	99.7 ± 0.9
2%/2 mm	91.7 ± 17.3	90.2 ± 15.6	95.9 ± 10.1
1%/1 mm	62.8 ± 21.2	53.9 ± 17.3	71.6 ± 19.5

**Table 5:** Gamma passing rates for the uncorrected (left) and new algorithm corrected (right) ArcCHECK measurements of 19 ViewRay patient plans

	Average gamma passing rates ± 1*σ*, %	
	Uncorrected	New algorithm corrected
3%/3 mm	96.9 ± 6.8	96.6 ± 7.2
2%/2 mm	84.0 ± 17.5	83.2 ± 18.9
1%/1 mm	49.1 ± 20.3	46.9 ± 19.6

**Table 6:** Average difference from ion chamber for ViewRay TPS, uncorrected, and new algorithm corrected ArcCHECK measurements at ten measurement points

	ArcCHECK uncorrected average difference from ion chamber	ArcCHECK new algorithm average difference from ion chamber
Difference in cGy	9.5 ± 3.5 cGy	9.6 ± 3.2 cGy
Difference in %	9.3 ± 3.4%	9.6 ± 4.0%

**Figure 9:**
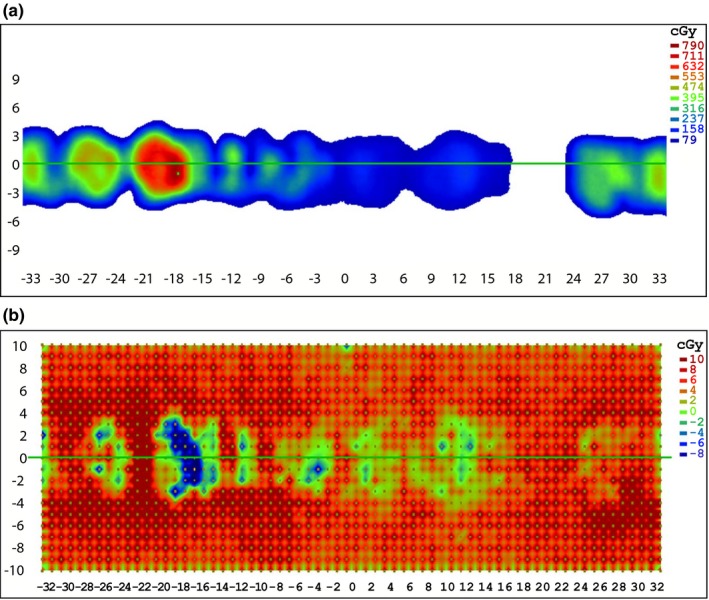
Screenshots from SNC Patient software of a dose map of a representative ViewRay treatment plan (a) and the corresponding dose difference map showing the differences between the ArcCHECK measurements and the treatment plan (b).

In Fig. 5, it can be seen that while applying the current correction factors with the new method seems to shift the discrepancy curve slightly closer to zero, the effect is very minor and there is still a significant positive offset. It can be seen in Table 4 that there is a significant difference between the gamma analysis results for the SNC corrected and new algorithm corrected datasets. This discrepancy is expected due to the difference in application of the factors combined with the ArcCHECK's discrepancies from the ViewRay TPS. The SNC algorithm is applied to every 50 ms measurement update and the results are summed for the total reading, while the new method weights the correction factors by the expected Monte Carlo‐calculated doses from each beam in a plan and applies the factors to the total uncorrected measurement.^2^ As Fig. 9 shows, there is a clear correlation between the calculated dose distribution and the corresponding map of differences between calculated and measured doses. This shows there is a difference between the calculated and measured relative dose distributions, which would cause the weighting of the correction factors to be different between the two methods and result in different final values.

In addition to the above results, the ArcCHECK corrections mode measurement showed similar frequency analysis results to the MATLAB implementation of the SNC algorithm, as shown in Fig. 6. All of the diode readings between the two datasets agreed within a global 1%. This indicates the SNC algorithm was properly implemented in MATLAB, with the slight differences between the two measurements attributable to the different heterogeneity and field size correction factors applied. By comparing Figs. 6(a) and 6(b), it can also be seen there is not a significant difference in the frequency distributions between ArcCHECK measurements performed in the normal ViewRay delivery mode compared to the Single Beam mode. This indicates leakage is not contributing significantly to the observed discrepancies between the ArcCHECK and ViewRay TPS.

Table 5 displays the gamma analysis results for applying the new algorithm on the original ArcCHECK measurements of the 19 ViewRay patient plans performed clinically. In contrast to the Single Beam Experiment, which consisted of a subset of the 19 patient plans, applying the corrections lowered the average gamma passing rates. These results were verified by repeating the original measurements for nine of the plans, with the repeated measurements matching these results. As the plans measured were the same as those used in the Single Beam Experiment, the same correction factors were applied. Using a two‐sided t‐test for dependent samples, the *P*‐value for differences between the uncorrected and new algorithm corrected datasets at 3%/3 mm is 0.33, and is not significant. However, at 1%/1 mm, the *P*‐value is 0.017. Applying the corrections therefore significantly lowers the average 1%/1 mm gamma passing rate. This observed difference between plans measured with the ArcCHECK with the ViewRay in Single Beam mode compared to the normal delivery mode indicates there is an additional effect not taken into account by the corrections.

**Table 7:** Additional uncertainties for ion chamber comparisons with uncorrected and corrected ArcCHECK measurements

Additional sources for dose difference comparisons	Uncertainty (%, k = 1)	Comments
ViewRay Monte Carlo TPS	1.0	Reported by ViewRay TPS
ArcCHECK Diode Accuracy/Consistency	0.8	Estimate based on variation between repeated ArcCHECK measurements
New correction algorithm beam weight extraction	2.0	Estimate based on average difference in extracted dose fraction between SNC Patient and MATLAB code

Diodes are known to give responses dependent on dose rate.^20^ While this may be a factor in the observed differences between the Single Beam and normal ViewRay deliveries, previous researchers have shown the ArcCHECK diodes to have only a ±1% dose rate dependence between approximately 20 and 600 cGy/min.^2^, ^3^ However, large magnetic fields may have an effect on this dependence. Additionally, there may be changes in angular response when multiple beams, each with an independent dose rate, are simultaneously incident on diodes. These possible dependencies will need to be investigated in the future.

**Figure 10:**
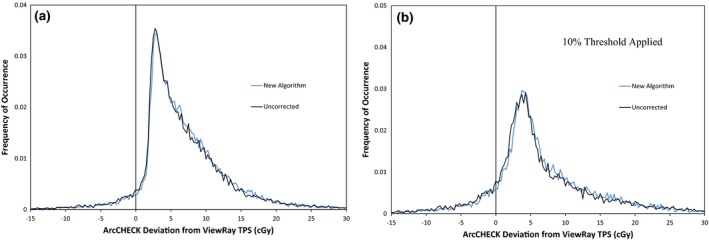
Frequency of occurrence vs. absolute difference in cGy between ArcCHECK (uncorrected and corrected with new algorithm) and ViewRay TPS for 26,334 ArcCHECK diode measurements across 19 ViewRay patient plans. Only measurements meeting a 10% global threshold are analyzed in (b).

The frequency plots and ion chamber comparisons show negligible difference between the corrected and uncorrected ArcCHECK measurements, as seen in Figs. 8 and 10. Only half of the corrected measurements were closer to the ionization chamber measurements than the uncorrected ArcCHECK measurements, and the improvements were very minor. Using a two‐sided t‐distribution, the calculated *P*‐value for the differences between the two sets is 0.73, and is therefore not significant.

From these results, it can be concluded that applying the current correction factors with either method does not consistently improve the accuracy of ArcCHECK measurements on the ViewRay system. As can be seen in Fig. 7, the correction factors are not designed to correct a systematic offset, such as what is observed for measurements on the ViewRay, as the average correction is near 1.00 for all of the factors. Additionally, using the current correction factors to apply the maximum possible reduction is only sufficient to correct a diode over‐response of 23%. It can be seen in Fig. 3 that this correction would not be adequate for approximately 10% of the ArcCHECK diode measurements meeting the 10% global threshold.

It has been shown that radiation dose deposition and diode response both change under the influence of an external magnetic field.^4^, ^5^, ^6^, ^7^ It can therefore be concluded the current correction factors, which were measured and/or modeled with Monte Carlo simulations without magnetic field effects taken into account, are invalid and/or inadequate for measurements on the ViewRay system.^2^, ^17^

In addition to the potential invalidity of the current correction factors, there also may be additional effects to the ArcCHECK diodes or electronics due to the magnetic field. If present, these effects need to be accounted for with additional correction factors and/or restructuring of the device.

Previous versions of the ArcCHECK required a repetition rate correction, as the diodes in the ArcCHECK gave responses dependent on the pulse frequency of the incident linear accelerator beams.^2^ There could be an additional effect with measurements on the ViewRay due to the ArcCHECK diodes and/or electrometer responding differently to the ViewRay's continuous ^60^Co beams compared to pulsed‐beam linacs. This potential dependency will need to be investigated in the future.

Despite these issues, it can be seen in Table 2 that the 19 ViewRay plans measured with the ArcCHECK have an average gamma passing rate greater than 95% at 3%/3 mm. Only three of the 19 plans have gamma passing rates lower than 95% at 3%/3 mm. The clinical significance of the observed discrepancies is therefore unclear. If the ArcCHECK is utilized solely to perform gamma analysis for patient‐specific quality assurance at 3%/3 mm, the discrepancies may not have a significant effect. However, for measurement procedures requiring higher accuracy and precision, such as those performed for TPS commissioning, the discrepancies would likely have a larger effect and lead to a greater uncertainty in delivered dose. Therefore, to be utilized in commissioning procedures with the ViewRay and other MR‐IGRT systems, further work must be performed to correct the observed discrepancies.

## 4 CONCLUSION

Using the ArcCHECK in the ViewRay environment consistently results in significant discrepancies between measured and planned doses. Applying the current correction factors does not consistently improve the ArcCHECK's accuracy for measurements on the ViewRay system. The current correction factors are therefore invalid and/or inadequate to correct the observed discrepancies between the ArcCHECK and ViewRay systems.

## ACKNOWLEDGMENTS

This work was funded by the Bhudatt Paliwal Professorship and the UWMRRC. The authors would like to thank the Sun Nuclear Corporation for the use of their phantom, and specifically thank Jakub Kozelka for the many discussions on this subject and for providing data and MATLAB code.

## CONFLICT OF INTEREST

The authors have no conflict of interest to report.

## Appendix A. derivation of eq. 3.

### A..1

According to Kozelka et al. and the Sun Nuclear Corporation's reference guide for the version of the ArcCHECK used at our institution, the dose to a single diode *i* for measurement update *j* is

(A1) $$D_{i,j}=N_{\mathrm{ref}}M_{\mathrm{corr},i,j},$$

where *N*_ref_ is the reading to dose calibration factor in Gy/reading and *M*_corr,i,j_ is the fully corrected diode reading.^2^, ^17^ The corrected diode reading is given by

(A2) $$M_{\mathrm{corr},i,j}=M_{i,j}C_{\mathrm{AD},i,j}C_{\mathrm{ID},i,j}C_{\mathrm{FS},i,j}C_{\mathrm{HF},i,j},$$

where *M*_i,j_ is the raw diode reading with background subtracted, *C*_AD_ is a factor correcting for the average angular dependence of the diodes for the given incidence angle, *C*_ID_ is the correction for individual deviations between the diodes for the given incidence angle, *C*_FS_ is the field size correction, and *C*_HF_ is termed the heterogeneity correction, which is used to match the diode response to an ion chamber measurement in a homogeneous PMMA phantom.^17^ The total dose to diode *i* for an entire measurement session is then

(A3) $$D_{\mathrm{TOT},i}=N_{\mathrm{ref}}\sum_{j=1}^{J}C_{i,j}M_{i,j},$$

where, for simplicity, all of the correction factors have been grouped into a single variable *C*_i,j_, and *J* is total number of measurement updates, given by the total measurement time divided by 50 ms/update. If we define a new variable *F*_i,k_ to represent the fraction of dose delivered to diode *i* by a specific fixed‐beam *k*, the total dose to diode *i* is also given by

(A4) $$D_{\mathrm{TOT},i}=\frac{D_{i,k}}{F_{i,k}}=\frac{N_{\mathrm{ref}}C_{i,k}M_{i,k}}{F_{i,k}}.$$

Also, the total raw measurement with background subtracted for diode *i* is

(A5) $$M_{\mathrm{TOT},i}=\sum_{k=1}^{K}C_{i,k}M_{i,k}=\frac{D_{\mathrm{TOT},i}}{N_{\mathrm{ref}}}\sum_{k=1}^{K}\frac{F_{i,k}}{C_{i,k}},$$

where K is the total number of fixed‐beams in the treatment plan. Combining eqs. 4 through 6 and rearranging, the total corrected dose to diode *i* is

(A6) $$D_{\mathrm{TOT},i}=N_{\mathrm{ref}}M_{i}\frac{1}{\sum_{k=1}^{K}\frac{F_{i,k}}{C_{i,k}}}.$$

Applying eq. 7 to the ViewRay ArcCHECK measurements, for which the calibration factor is already applied, the corrected ArcCHECK diode dose is

(A7) $$D_{\mathrm{TOT},\mathrm{corr},i}=\frac{D_{\mathrm{TOT},\mathrm{uncorr},i}}{\sum_{k=1}^{K}\frac{F_{i,k}}{C_{\mathrm{AD},i,k}C_{\mathrm{ID},i,k}C_{\mathrm{FS},i,k}C_{\mathrm{HF},i,k}}}.$$
